# More than Just a Meal: Breakfast Club Attendance and Children’s Social Relationships

**DOI:** 10.3389/fpubh.2015.00183

**Published:** 2015-07-30

**Authors:** Margaret Anne Defeyter, Pamela Louise Graham, Riccardo Russo

**Affiliations:** ^1^Department of Psychology, Northumbria University, Newcastle upon Tyne, UK; ^2^Department of Psychology, University of Essex, Essex, UK

**Keywords:** breakfast clubs, friendship, peer victimization, children, social relationships

## Abstract

The health benefits of school food have been widely promoted in recent years while the social opportunities that surround eating occasions at school have received little attention. Breakfast clubs (BCs), which take place at the start of the school day, offer a unique opportunity for children to consume a breakfast meal on their school premises in the company of their peers. Alternatively, after-school clubs (ASCs), which take place on school premises at the end of the school day, whilst also providing children with social opportunities tend to focus on sports engagement and skill development. The aim of the current paper is to investigate whether attendance at BCs and ASCs has an impact on children’s friendship quality and experiences of peer victimization. BC attendees, ASC attendees, and non-attendees completed the Friendship Qualities Scale and the Multidimensional Peer Victimization Scale (MPVS) at two time points. Time-1 data were collected 2 months after the introduction of school clubs. Time-2 data were then collected on the same measures again 6 months later. Results of the analyses of Time-1 data showed no significant differences between groups on any of the measures at Time-1. However, at Time-2, BC attendees showed improved levels of friendship quality compared to the other two groups. Moreover, analysis of the MPVS data at Time-2 showed that children who attended BC or ASC experienced a decline in victimization across time. The current findings suggest that BC attendance facilitates the quality of children’s relationships with their best friend over time. Additionally, attendance at a breakfast or ASC was associated with a reduction in victimization over time. The results have implications for utilization of breakfast and ASCs to aid children’s social relationships in school over time.

## Introduction

The benefits of school food provision on children’s health, cognitive performance, and academic attainment have been highlighted across research and policy (1–4). However, it is scarcely acknowledged that eating occasions that take place on the school premises tend to occur in social settings thus offering children unique opportunities to socialize with peers whilst consuming a meal (5). Research has shown that eating a meal alongside others can facilitate interaction amongst individuals (6) and offer occasions in which to teach children social skills (7). This is particularly important because relationships have been found to have a substantial influence on behavior and numerous developmental outcomes throughout childhood and adolescence (8) including academic performance (9, 10), self-confidence (11), and attitudes toward school (12). However, to have a more in-depth understanding of the impact of friendships on children’s emotional well-being, it is essential to take into account the quality of children’s friendships (13) and not simply the presence or absence of friends.

A high quality friendship is typically characterized by high levels of positive features such as companionship, help, security, and closeness and low levels of conflict (14). Taking into account the multifaceted nature of friendships, research has shown that friendship quality is an important predictor of overall emotional well-being and loneliness (13, 15), young children’s early school adjustment (16), and the development of interpersonal sensitivity and emotional security (17).

More specific research on the impact of friendships on children’s well-being has shown that, as well as encouraging positive emotional and academic outcomes, friendship can also act as a protective factor against peer victimization (18–20). Victimization refers to being a recipient of any kind of aggressive attack and is usually measured, unlike bullying, without reference to the power imbalance between the perpetrator and the victim or to the repetitive nature of the act. Four types of victimization have been identified: physical victimization, verbal victimization, social manipulation, and attacks on property (21, 22).

It has been suggested that the display of certain emotional and behavioral characteristics including anxiety, self-focused behaviors, and poor social skills can make children more susceptible to peer victimization (23). Similarly, it has been shown that children with poor social skills report lower quality friendships, which in turn increase their vulnerability to victimization (24).

Play has been described as the “central medium of social interaction with peers in early childhood” (25) (p. 143). Through play and shared activities, children learn an array of skills necessary for successful social interactions with peers (26). It is therefore plausible to argue that it is crucial to allow children to spend time playing with peers in order to allow them to develop favorable social abilities. However, the number of play opportunities available to children in schools has decreased dramatically in recent years, particularly because a greater emphasis has been placed on academic activities and school break times have been markedly reduced (27, 28).

Despite this reduction in break times during the formal school day, schools have increased the number of groups and activities that they offer to pupils and their families outside of the school day. In 2010, the Department for Education reported that 98% of schools in England were providing access to extended services including breakfast and after-school clubs (ASCs) for children, support groups for parents and families, and adult education classes (29). The provision of breakfast and ASCs hold particular significance as the activities that children partake in outside of school hours have been found to impact upon numerous developmental outcomes. For example, research suggests that adult-supervised care in after-school activities is more favorable for children than self-care outside of school hours, as it has been associated with positive peer relationships, better emotional adjustment, and better conduct (30). On the contrary, self-care has been linked to behavioral problems such as aggression and defiance (30). Furthermore, before and ASCs potentially offer children opportunities to spend time with their peers at a time when play opportunities are continually being reduced (31).

School breakfast clubs (BCs) typically offer children the opportunity to consume a nutritious breakfast in the company of school staff and peers on the school site before going into class (32). Moreover, unstructured, face-to-face interactions with peers within small groups usually occur during the BC hour prior to school time. An increase in the number of BCs available in the UK in recent years has been driven in part by the expansion of extended school services (33) but also through a growing awareness of the detrimental effects of skipping breakfast (34, 35).

After-school clubs take place at the end of the formal school day and allow children to partake in structured activities including sports, academic subjects, and performing arts (36) whilst under the supervision of non-familial adults (37). It has been proposed that children who are involved in activities are seen as valued members of the school community (38).

Although BCs and ASCs share similar features in that they offer children opportunities for regular participation and staff direction (32, 39), it appears that there are differences in the key foci of each club. BCs generally aim to provide children with the opportunity to consume a nutritious breakfast on school premises before the start of the school day (40). Moreover, they offer the opportunity for unstructured face-to-face interactions between children to occur (41). In comparison, ASCs aim to provide children with opportunities for skill acquisition and development (39, 42, 43).

In meeting their different aims, BCs and ASCs both offer children the same unique opportunity to spend time with peers they might not otherwise be able to spend time with (31). Despite this, the potential impact of attendance at BCs and ASCs on children’s peer relationships has generally been overlooked within the research literature. This is surprising given that time spent with peers is thought to be a major contributor to children’s social development (44) and children’s social abilities have the potential to influence the quality of their friendships and experiences of victimization (23, 24).

Given this background, the aim of the current study is to evaluate the impact of children’s attendance at BCs and ASCs on the quality of their relationships with their best friend and their experiences of peer victimization. This is the first study to examine the potential relationship between these factors. Gender differences will also be evaluated in the present study as gender differences in victimization are not obvious (46). As mentioned previously, BCs and ASCs both offer children the opportunity to spend additional time with their peers outside of school time but the nature of activities available to children within these clubs are different. We would therefore tentatively predict different outcomes for friendship quality and peer victimization dependent on the type of club that children attend. BCs focus predominantly on the provision of a breakfast meal where children are involved in informal, dyadic, or small group interactions with no pursuit of achievement. For this reason, it is possible to predict that involvement in BCs could potentially benefit the perceived quality of children’s dyadic relationships. ASCs on the other hand are generally more oriented toward sports and competitive activities involving relatively large groups of children. Given that activity participants are recognized as valued members of their school communities (38), we would expect ASC attendance to be linked to decreased levels of peer victimization. To assess the above hypotheses, in the present study, we measured both quality of the relationships of children with their best friend [Friendship Qualities Scale (FQS) (45)] and their experiences of peer victimization [Multidimensional Peer Victimization Scale (MPVS) (22)] in self-selected samples of children who either attended BCs, or ASCs, or neither breakfast nor ASCs.

## Materials and Methods

### Design

The current study utilized a 3 × 2 × 2 mixed factorial design with club membership [BC; ASC; None (NC)] and gender treated as between-subjects factors and time (Time-1; Time-2) as a within-subjects factor. Time-1 data collection took place 2 months after the start of the academic year (November 2009) and Time-2 data collection took place 6 months later (April 2010).

### Participants

Two hundred and eighty-five primary school children were recruited in total from eight mixed gender primary schools in the UK. Ethical approval for this study was granted by Northumbria University Ethics Committee. Fully informed consent was obtained from all Head Teachers, parents, and pupils. All of the schools were inner-city community schools with a catchment area of pupils from predominantly white, lower socio-economic status families. Five children failed to participate in the second phase of testing and two children stopped attending BC, so their data were removed from subsequent analyses. In addition, 10 children (4 children from the BC group, 4 from the ASC group, and 2 from NC) could not name a “best” school friend and their data were excluded from analyses. Hence, analyses were conducted on 268 primary school children (mean age = 8.4 years, SD = 1.69, range 5.3–10.11 years); 163 females (mean age 8.2) and 105 males (mean age 8.5). Participating children were then further divided into the following three groups: BC attendees (i.e., BC; *N* = 94), non-club-attendees (i.e., NC; *N* = 88), and ASC attendees (ASC; *N* = 86) according to the following criteria. BC attendees attended a school BC a minimum of two sessions per week, and attended no other after-hours school club. All BCs were school-led and did not receive any sponsorship from external organizations. BCs served children with cereals, toast, fruit, and yogurt, which were purchased from local supermarkets by BC staff. BCs also allowed children to partake in structured indoor activities such as table-top games and drawing. ASC attendees attended an ASC a minimum of two sessions per week and did not attend BC. All of the ASCs were sport/physical activity clubs (football clubs, gymnastics, or mixed sporting activity clubs). Children in the no club group attended neither a BC nor an ASC. Observation of the demographic data shown in Table 1 showed that groups were comparable with one another in terms of ethnicity and socio-economic class. Details regarding the composition of the clubs can be seen in Table 2.

**Table 1:** **Sample characteristics**.

	School breakfast club attendees	After-school club attendees	No club
**Gender**
*N*	94	86	88
Male	35	39	31
Female	59	47	57
**Age in years**			
Mean	8.24	8.75	8.24
SD	1.60	1.58	1.85
**Ethnicity (%)**			
White	94	95	94
Black	1	1	2
Asian	5	4	3
**Eligible for free school meals (%)**			
Yes	71	78	74
No	29	22	26
**Occupation of head of household (%)**			
Professional	3	5	2
Managerial/technical	10	8	7
Skilled non-manual	4	2	3
Skilled manual	52	49	53
Not working	31	36	35
**Days of school skipped within last month (% taken from school records)**			
None	90	88	92
One or more	10	12	8

**Table 2:** **Composition of school clubs**.

School	1	2	3	4	5	6	7	8
**OVERALL FOR SCHOOL**								
Total number of pupils on roll	222	309	320	348	324	248	215	309

% of pupils entitled to free school meals	71	74	72	74	68	66	66	71

**BREAKFAST CLUB GROUP**								
Number of pupils attending	24	26	31	29	18	26	12	32

Staff:student ratio	1:12	1:13	3:31	2:29	1:6	1:13	1:6	1:16

Duration of club each day	45 min	60 min	55 min	45 min	50 min	45 min	60 min	40 min

Club environment	School dining hall	Classroom	School dining hall	Classroom	School dining hall	School dining hall	School dining hall	School dining hall

Type of activities	Breakfast, board games, reading	Breakfast, computer games, board games, coloring	Breakfast, board games, coloring, reading	Breakfast, computer games, coloring, singing	Breakfast, board games, coloring	Breakfast, coloring, reading, construction games	Breakfast, board games, coloring	Breakfast, coloring, construction games (e.g., Lego)

Number of pupils in study sample	7	6	11	22	14	7	12	15

Mean age (SD)	8.0 (1.34)	8.4 (1.2)	7.8 (1.75)	7.5 (1.36)	8.4 (1.20)	7.5 (1.10)	8.2 (1.00)	7.7 (1.10)

Min–max	6.5–9.11	7.0–9.7	5.3–10.11	5.4–10.4	6.8–9.10	6.4–9.1	7.0–9.6	6.4–9.2

**AFTER-SCHOOL CLUB GROUP**								
Number of pupils attending	18	26	28	17	33	31	22	28

Staff:student ratio	1:9	1:13	1:14	3:17	1:11	2:31	1:11	1:14

Duration of club each day	60 min	45 min	60 min	75 min	60 min	45 min	60 min	60 min

Club environment	Sports hall	Football field	Football field/play ground	Sports hall/football field	Football field	School hall	Classroom/play ground	Classroom/play ground

Type of activities	Physical activity games	Football	Football, physical activity games	Physical activity games, football	Football	Indoor play activities (Hoopla and organized games)	Homework, organized play activities	Homework, free outdoor play activities

Number of pupils in study sample, *N* = 86	12	15	11	7	9	14	8	10

Mean age (SD)	8.4 (1.2)	8.7 (1.6)	7.5 (1.0)	8.7 (1.2)	7.8 (0.6)	8.0 (1.3)	8.6 (1.1)	8.3 (1.2)

Min–max	6.9–10.6	6.4–10.2	6.3–9.5	6.7–10.2	7.5–8.3	5.5–9.11	6.9–10.4	6.4–10.8

**NO CLUBS GROUP**								
Number of pupils not attending a school club	180	257	261	302	273	191	181	249

Number of pupils in study sample, *N* = 88	9	7	9	18	13	14	10	8

Mean age (SD)	8.2 (0.97)	8.5 (1.02)	8.2 (0.41)	8.3 (0.7)	7.9 (1.3)	8.2 (0.87)	8.5 (1.00)	7.6 (1.08)

Min–max	6.5–9.11	7.7–10.2	6.1–9.7	8.0–9.2	6.5–9.7	7.3–9.5	7.0–10.11	5.7–10.3

### Measures

#### Friendship Qualities Scale

Pupils completed an adapted version of the FQS (45). The FQS represents five dimensions of friendship: companionship, conflict, help, security, and closeness. Companionship is represented by a set of items which focus on the amount of voluntary time spent together. Conflict is represented by a set of items which examine whether the child gets into fights, arguments, or disagreements with their friend. Help is represented by two subscales: (a) aid, which considers the features of mutual help and assistance within the friendship and (b) protection from victimization, which explores a friend’s willingness to assist the child if they were to be bothered by another peer. Security is measured by items that focus on the belief that (a) the friendship can withstand an argument and (b) friends can be relied upon during times of need. Finally, closeness focuses on the sense of affection that the child experiences with a friend and the strength of the child’s attachment to the friend. Each dimension is made up of a series of simple statements (e.g., “My friend would help me if I needed it”) and children are asked to rate how true they perceive each sentence to be about their relationship with their best friend. Children rate each statement on a five-point scale ranging from 1 (“not true at all”) to 5 (“really true”). Cronbach’s Alpha of the FQS (range = 0.71–0.80) are reported to be acceptable (47).

In addition to completing the FQS, pupils were asked to name their “best” friend. As an opt-in method of consent was used, some children were excluded from participating in the study due to lack of parental consent, so it was not possible to consider reciprocal friendship nominations. Therefore, in order to verify whether named children were best friends with the participant; teachers were subsequently asked to confirm whether pupils were, indeed, best friends or simply friends.

#### Multidimensional Peer Victimization Scale

Anonymous self-report is said to be the most reliable method of measurement for peer victimization data collection (22). Pupils therefore completed a version of the MPVS (22). The MPVS represents four dimensions of victimization: physical victimization, social manipulation, verbal victimization, and attacks of physical property. The physical victimization subscale looks at how often children have been subjected to physical harm such as being punched or kicked. The social manipulation scale is concerned with incidents involving negative social behavior, for example persuading other children not to talk to a particular child. The verbal victimization scale examines spoken behaviors such as name calling. Finally, the attacks of physical property scale investigates victimization through the damage or theft of possessions. Scores on the total scale have a possible range of 0–32 and a possible range of 0–8 on each of the four subscales. A higher score indicates that a child has been subjected to more incidents of peer victimization. Internal consistency (range = 0.73–0.85) and criterion validity of the scale are reported to be acceptable (48).

For the purposes of the present study, the questionnaire recorded levels of victimization experienced during the 6-month study period between Time-1 and Time-2. To check that children understood the questions and to make sure that incidents were not just examples of rough and tumble play, children were asked to provide examples to each question.

### Procedure

Opt-in parental consent was obtained for all children prior to participation. Children completed the questionnaires, as described in the Section “Measures,” 2 months after the start of the academic year (i.e., Time-1; November 2009) and were then tested again on the same measures, approximately 6 months later (i.e., Time-2; April 2010). By Time-2, those children attending either BC or ASC had been doing so for a period of 8 months. All questionnaires were completed in a quiet area of children’s normal classrooms, with the researchers present. For children below the age of eight, the researchers read out the questions. Data were also collected from teachers at Time-1 and Time-2.

### Structure of analysis

The data obtained from the FQS and from the MPVS were analyzed in the following manner. Firstly, a one-way between subjects analysis of variance (ANOVA) was performed on the scores of each subscale at Time-1 to assess if the groups (BC only, i.e., BC; ASC only, i.e., ASC; and No club, i.e., NC) were comparable at Time-1. Eventual significant results were followed-up by Tukey HSD test.

Then, factorial ANOVAs where club membership (BC, ASC, and NC) and gender were between-subjects factor and Time (Time-1 vs. Time-2) was a within-subjects factor were performed on each subscale. Since no two-way interaction involving the time factor was significantly qualified by the gender factor, data were collapsed over genders. Hence, the reported analyses are from two-factor ANOVAs where the gender factor was omitted.

To follow-up any significant Group by Time interaction, difference scores, on the basis of no significant differences between conditions at Time-1, were computed between the measurements at Time-2 minus Time-1, then 99% confidence interval were constructed for each Group (99% CI were chosen to keep the risk of a type I error in each family of comparisons at 0.05).

SPSS version 19 was used to analyze the data.

## Results

### Friendship qualities scale

Children were very accurate at naming school friends; but teacher–pupil agreement was not so strong for “best” friends. There was 100% agreement by teachers regarding children’s naming judgments at both Time-1 and Time-2 in terms of friendship but only 68% agreement at Time-1 and 65% agreement at Time-2 in terms of “best” friend judgments. Overall, 94% of children named same sex friends; and teacher–pupil agreement on “best” friend naming did not significantly differ across groups. Hence, no further children than those indicated in the Section “Materials and Methods” were removed from subsequent analyses.

The statistical analyses reported were conducted on the data shown in Table 3. The one-way ANOVAs on Time-1 scores did not show any significant difference between groups in any of the subscales, *F*s (2, 265) < 1.16, *p* > 0.10, thus suggesting that the performance of the three groups of pupils were comparable at Time-1. The main outcome of the factorial ANOVAs was the presence of significant groups by time interactions for each subscale. As written above, these were followed-up by comparing the 99% CI on the difference scores between performance at Time-2 minus Time-1 among the three groups given that Time-1 data were comparable among groups of pupils. These 99% CI as well as the mean difference scores for each group and each subscale are displayed in Table 3.

**Table 3:** **Section 1: performance at Time-1 and Time-2 in the FQS in the three club groups, section 2: the mean of the difference scores Time-2 − Time-1^a^**.

		BC	ASC	NC
**SECTION 1**				
Closeness	Time-1	4.07	3.95	4.11
	Time-2	4.39	3.59	3.85
Conflict	Time-1	3.40	3.43	3.42
	Time-2	3.10	4.07	3.90
Companionship	Time-1	3.79	3.91	3.88
	Time-2	4.23	3.79	3.69
Help	Time-1	4.09	3.97	4.04
	Time-2	4.41	3.64	3.59
Security	Time-1	3.64	3.78	3.80
	Time-2	4.02	3.52	3.65
**SECTION 2**				
Closeness	Mean	0.322	−0.352	−0.263
	99% CI	0.156 to 0.487	−0.525 to −0.179	−0.434 to −0.092
Conflict	Mean	−0.295	0.642	0.483
	99% CI	−0.520 to −0.071	0.408 to 0.877	0.251 to 0.715
Companionship	Mean	0.436	−0.122	−0.188
	99% CI	0.283 to −0.590	−0.283 to 0.038	−0.347 to −0.029
Help	Mean	0.316	−0.328	−0.455
	99% CI	0.124 to 0.508	−0.530 to −0.128	−0.653 to −0.256
Security	Mean	0.380	−0.253	−0.151
	99% CI	0.198 to 0.563	−0.444 to −0.062	−0.339 to 0.038

*^a^99% CI are provided for each subscale at each group level. This level of significance was adopted to keep the family wise error rate at 0.05 within the five sets of analyses for the Friendship Qualities Scale*.

As it appears in the lower part of Table 3, the 99% CI for the BC group never overlapped with those of the remaining groups, while the CI for these remaining groups overlapped for every subscale used. Overall, this indicated that the children attending BC groups differed significantly in the scores between Time-2 and Time-1 compared to the ASC and the No Club Group. Below, a more complete summary of the results is provided.

For the *Closeness subscale*, the significant interaction between time and group type [*F* (2, 265) = 31.94, MSE = 0.192, *p* < 0.01, = 0.194] was because of a significant increase in the level of closeness in the BC group at follow-up, while there was a significant decrement in the other groups.

For the *Conflict subscale*, the significant interaction between time and group type [*F* (2, 265) = 32.62, MSE = 0.352, *p* < 0.01, = 0.198] occurred because the level of conflict in the BC group lowered significantly at Time-2, while in the other two groups it increased significantly.

For *Companionship*, the significant interaction between time and group type [*F* (2, 265) = 32.74, MSE = 0.164, *p* < 0.01, = 0.198] was characterized by a significantly higher level of companionship at Time-2 in the BC group compared to the other two groups where, at least in the no clubs group, significantly lower levels of companionship where detected.

In terms of the *Help subscale*, the significant interaction between time and group [*F* (2, 265) = 30.40, MSE = 0.258, *p* < 0.01, = 0.187] occurred because the perceived level of help that children perceived they receive from their friend increased significantly at Time-2 compared to Time-1 in the BC group compared to the other two groups where it decreased significantly.

Finally, in terms of the *Security subscale*, the significant interaction between time and group type [*F* (2, 265) = 22.70, MSE = 0.232, *p* < 0.01, = 0.146] could be accounted for by a significant increase in the level of security in the BC group unlike the other groups where there was either no significant change in the level of security between Time-1 and Time-2 (the NC group) or there was a significant reduction in the level of security (the ASC group).

### Multidimensional peer victimization scales

The statistical analyses reported were conducted on the data shown in Table 4. The one-way ANOVAs on Time-1 scores did not show significant differences across groups for social, verbal, and attacks of property subscales, *F*s (2, 265) < 1.7, *p* > 0.10, thus suggesting that the performance of the three groups of pupils were comparable at Time-1. The only significant difference occurred in the Physical Victimization subscale, *F* (2, 265) = 3.92, *p* < 0.05. However, Tukey HSD *post hoc* analyses showed no significant differences between pairs of means.

**Table 4:** **Section 1: performance at Time-1 and Time-2 in the PVS in the three club groups, section 2: the mean of the difference scores Time-2 − Time-1^a^**.

		BC	ASC	NC
**SECTION 1**				
Physical	Time-1	2.904	2.477	2.375
	Time-2	1.872	1.407	2.057
Social	Time-1	3.043	2.686	2.716
	Time-2	2.053	1.872	2.420
Verbal	Time-1	3.096	3.000	3.034
	Time-2	2.660	2.488	2.705
Property	Time-1	1.053	0.872	1.114
	Time-2	1.106	0.895	0.659
**SECTION 2**				
Physical	Mean	−1.032	−1.07	−0.318
	99% CI	−1.365 to −0.699	−1.418 to −0.722	−0.662 to 0.026
Social	Mean	−0.989	−0.814	−0.295
	99% CI	−1.368 to −0.611	−1.21 to −0.418	−0.687 to 0.096
Verbal	Mean	−0.436	−0.512	−0.33
	99% CI	−0.802 to −0.071	−0.894 to −0.13	−0.707 to 0.048
Property	Mean	0.053	0.023	−0.455
	99% CI	−0.287 to 0.393	−0.333 to 0.379	−0.806 to −0.103

*^a^99% CI are provided for each subscale at each group level*.

The main outcomes of the factorial ANOVAs were the following: for the *Physical Victimization subscale*, there was a significant interaction between time and group type [*F* (2, 265) = 10.26, MSE = 0.773, *p* < 0.01, = 0.072]. This occurred because of the significantly lower level of physical victimization in the BC and ASC groups at Time-2 compared to Time-1, while in the NC group levels of physical victimization did not differ significantly between Time-2 and Time-1.

Likewise, there was also a significant interaction between time and group type for *Social Manipulation subscale* [*F* (2, 265) = 5.84, MSE = 1.001, *p* < 0.01, = 0.042], with significantly lower level of social manipulation in the BC and ASC groups, while in the NC group levels of social manipulation did not differ significantly between Time-2 and Time-1. Although there were lower levels of *Verbal victimization* at Time-2 compared to Time-1 [*F* (1, 265) = 26.02, MSE = 0.932, *p* < 0.01, = 0.089], there was no significant interaction between time and group type [*F* (2, 265) < 1]. Finally, for the *Attacks of property subscale*, there was a significant interaction between time and group type [*F* (2, 265) = 4.46, MSE = 0.809, *p* = 0.012, = 0.033], with the only significant difference occurring in the NC group; suggesting that attacks on property decreased across time only for the NC group.

## Discussion

The current study aimed to evaluate the impact of breakfast and ASC attendance on children’s self-reported friendship quality and peer victimization. Overall, the obtained results partially confirmed the predictions made in the introduction. More specifically, BC attendance had a positive impact on the quality of children’s friendships with their best friend. Furthermore, both BC attendees as well as ASC attendees reported significantly reduced levels of peer victimization over a 6-month period.

Children’s peer relationships have been found to have a significant effect on numerous developmental outcomes including social competence (49, 50) and academic achievement (9, 10). One key aspect of friendship is thought to be companionship, which refers to the amount of voluntary time friends spend with one another (45). Given that many children spend free time with their peers in BCs and ASCs, it is surprising that the potential impact of attendance at these clubs on children’s peer relationships has received little attention in the research literature.

Analyses of the FQS showed that after 6 months, children attending BC reported higher levels of companionship, closeness, help, and security compared to the ASC group and the no clubs group, which showed the opposite pattern. These findings are particularly striking given that children in the BC group and the ASC group spent a similar amount of time in adult-led activities. The current study also found reduced levels of conflict, at Time-2, in the BC group compared to the other two groups where conflict increased. The results obtained suggest that the quality of children’s friendships is comparable for all groups at the start of the school year but for those children who do not attend BC, the quality of their friendships decreases. It may be the case that children’s friendships are positive at the start of the school year because they have all just returned to school after a long summer break and are therefore happy to be spending time with friends they missed during the school holidays. As the school year progresses and children spend more time together, there are more opportunities for conflicts to arise and this might result in a reduction in friendship quality as the initial excitement of returning to school and seeing friends dissipates. The pattern of results obtained suggests however that attendance at BC might prevent this deterioration in friendship quality and actually lead to improvements in children’s dyadic relationships.

A key difference between BCs and ASCs is that BCs offer children opportunities for unstructured, face-to-face interaction with peers within small groups during the breakfast meal. It has been established that family meal times facilitate interaction amongst family members (51) and offer a unique opportunity to teach children social skills (52). One possibility is that the small, unstructured group interactions that take place during the breakfast meal within BC also help children to develop social skills, enabling them to create meaningful and close friendships and resolve conflict. Given that the breakfast meal is the predominant feature of BC, further investigation is required to evaluate the specific impact of breakfast meal interactions that take place in BC. In summary, against a background of deteriorating friendship quality over time among the groups that either did not attend any school club or attended an ASC, the present findings suggest that BC attendance is associated with overall improvements in the quality of dyadic friendships. Finally, analysis of the friendship quality data revealed that none of the significant two-way interactions between the group type and the time of test were qualified by gender, thus it seems that the impact of attending BC is comparable in male and female children.

Much debate surrounds the question of how best to measure children’s friendships and particularly whether reciprocal friendship nominations are essential (13, 53). The design of the current study meant that it was not possible to collect pupil-led data on whether friendships were reciprocal; however most children were able to name a “best friend” whom attended the same school, and the reciprocal nature of this relationship was verified by the children’s primary teacher. Reciprocity in children’s friendships is difficult to clarify. Although teachers and parents are often believed to be well placed to identify friendship groups, disagreement between child and adult reports on peer interactions have been found as early as pre-school (31). The 100% agreement between pupils and teachers regarding children’s friendship nominations in the current study suggests that participating teachers were familiar with the peer groups under investigation. The 65–68% agreement between teachers and pupils on best friend nominations is also highly acceptable.

Analyses of the MPVS data provide a more mixed pattern of results. Overall, levels of physical, social, and verbal victimization decreased over time; while the level of attacks on property remained constant. Eight months following the introduction of school BCs, children in the BC and in the ASC groups reported lower levels of physical victimization compared to the no clubs group. Moreover, at the second test time, decreased levels of social victimization and attacks on property were found both in the BC group and the ASC group. The results also showed that gender did not qualify any group type by time interaction, thus the present study adds to the growing number of studies that have reported no gender differences in terms of victimization (27).

Overall, these findings suggest that participation in structured activities before- and after-school help in reducing children’s experiences of peer victimization. Involvement in activities can help children to become more resilient, i.e., better able to deal with stressful situations (54). Moreover, it has been suggested that when children engage in play, they develop skills in conflict resolution and emotion regulation (25). Bringing these ideas together, it might be the case that the children who attended BCs and ASCs in the current study had developed positive social skills through play and interaction with other children and adults and this in turn allowed them to behave in a more favorable manor toward their peers and thus avoid being victimized. This explanation would also fit with ideas proposed by Bierman (23) who suggested that victimized children often behave in a way that encourages a cycle of victimization.

Theories surrounding the protective element of friendship offer a further potential explanation for the observed reduction in victimization in the BC group and the ASC group. According to Vitaro et al. (55), “friends may protect against the negative consequences of peer victimization by providing companionship, emotional support, intimacy, and self-validation” (p. 572). It may be that children who attend clubs outside of school time have more opportunity to experience the protective provisions offered by friendship than pupils who attend no clubs.

The finding that breakfast and ASCs impact on levels of victimization in primary school children warrants further investigation. Research has shown that more cases of physical, verbal, and indirect victimization by peers at school are reported by younger students than by students in higher grades (55–59). Whilst recognizing that further work is required to identify particular features of breakfast and ASCs that impact on peer victimization, such as the ratio of staff to pupils, the preliminary findings of this research are encouraging. Especially when one considers that research has shown schools with large populations of pupils from families with low socio-economic status typically have higher levels of indirect victimization (60). However, some researchers have pointed out that peer victimization does not only occur in the classroom and playground, but may occur on the way to and from school (60). Hence, an alternative hypothesis is that differences in levels of victimization between groups is not a consequence of attending breakfast or ASCs *per se*, but rather differences in modes of transport to and from school. Researchers have suggested that different modes of transport to and from school may impact on levels of victimization (60); and it may be the case that children are more likely to be dropped off and picked up by parents if they attend a breakfast or ASC thus reducing their potential for exposure to victimization.

Researchers have emphasized the importance of developing a positive school climate to reduce school violence, such as positive student–teacher relationships, and student participation in decision making and policies (61–65). It may be the case that BC attendees and ASC attendees have a more positive perspective of the school, better relationships with teachers, and may feel more integrated into the school’s community compared to non-attendees. Future research is planned to evaluate potential differences in pupils’ perceptions of their school climate.

## Strengths and Limitations

Although care was taken to read the questions to young children and check all children’s understanding by asking for examples, there remains the possibility that not all children fully understood the questions. It has been suggested that developmental limitations in the verbal and cognitive comprehension skills of young children might prove problematic in using self-report methods to investigate peer relationships (26). However, even if this was the case, it is expected that these problems would be present at a comparable rate among the youngest children in all tested groups. This may add a relatively constant noise in the data of the three groups. Hence, the results observed are unlikely to be influenced by the potential presence of a small constant bias in the three groups.

Finally, recent researchers investigating the impact of school interventions including BCs and ASCs have acknowledged that it is not always plausible to conduct randomized controlled trials in these cases, despite this method being considered the gold standard in evaluative projects (66). Questions such as whether it is ethical to prevent a control group of children from attending a potentially beneficial intervention and whether it is possible to maintain strict control and treatment groups for the duration of the evaluation have been raised (33, 67, 68). Due to the nature of the independent variable employed in the current study, it was not possible to conduct a randomized control trial on ethical grounds. Implementation of a randomized controlled trial in this study would have resulted in some children being prevented from attending a club where a meal was provided and it might have been the case that some children chose to attend the BC in order to get a breakfast meal because it was not possible for them to have breakfast elsewhere. However, it should be noted that there were no significant differences between groups on any of the measures taken at Time-1. Hence, suggesting that, irrespective of the reasons for either attending or not attending a school club, the three groups appeared to show comparable Time-1 performance in the scales used. Therefore, any significant differential change over time in the dependent variables across groups cannot be ascribed to differences across groups at Time-1.

In conclusion, the present study has provided the first empirical evidence that attendance at BC can improve the perceived quality of dyadic friendships, which otherwise seems to be perceived as significantly deteriorating over time as it appeared to occur among the children who did attend ASCs or no clubs. BCs are often utilized by parents as a means of before-school childcare provision and it has been suggested that BCs offer children a unique opportunity to interact with adults, children of other age groups, and friends in a calm environment (41). In comparison, after-school activities are generally attended by children who have an intrinsic interest in the activity taking place (39) and those who are more competent at the activity (43). There is a possibility that the non-competitive nature of the BC environment helps to facilitate children’s relationships because there is no focus on achievement or competition between children. Further research is required to examine whether the activities taking place at BC help to foster a more balanced, higher quality relationship than after-school activities. Furthermore, attendance at school clubs seems to be associated with reduced levels of peer victimization. Hence, the present study provides further evidence of the potential for extra-curricular, semi-organized school activities to provide children with the necessary support and skills to protect them from victimization.

## Conflict of Interest Statement

This research was funded by Kellogg’s.
